# Adsorption and Desorption of Bioactive Proteins on Hydroxyapatite for Protein Delivery Systems

**DOI:** 10.1155/2012/932461

**Published:** 2012-03-05

**Authors:** Chie Kojima, Kenji Watanabe

**Affiliations:** ^1^Nanoscience and Nanotechnology Research Center, Research Organization for the 21st Century, Osaka Prefecture University, 1-2 Gakuen-cho, Naka-ku, Sakai, Osaka 599-8570, Japan; ^2^Department of Applied Chemistry, Graduate School of Engineering, Osaka Prefecture University, 1-1 Gakuen-cho, Naka-ku, Sakai, Osaka 599-8531, Japan

## Abstract

Hydroxyapatite (HA) is a precursor of bone and has been studied as a biomaterial. We attempted HA to apply to protein delivery systems. In this study, the association and dissociation properties of two types of bioactive proteins, cytochrom c and insulin, to HA were investigated. Cytochrom c was less associated with HA than insulin, which was easily released from it. However, the release of insulin from HA was slow. Insulin was released from HA at pH 7.4 more rapidly than at pH 3. The association and dissociation properties might be influenced by the size, solubility and net charge of protein. HA is a potential protein carrier with controlled release.

## 1. Introduction

Drug delivery systems (DDSs) are useful for reducing drug side effects and maximizing drug action. The design of drug carriers for DDS is the most important activity in this area. The advent of molecular biology studies has enabled the identification of many disease-causing proteins. Because some of these are effective as drugs, protein delivery systems have become important in DDS. A variety of nanoparticles such as liposomes, micelles, and polymers have been adopted as drug carriers [1–4]. Because polymers are similar in size to proteins, they are not suitable as protein carriers. Whereas liposomes and micelles are larger than polymers and proteins, they can be used as protein carriers. However, because these are self-assembled nanoparticles, some treatments are necessary for their preparation as carriers. Since most proteins are sensitive to temperature, pH, and organic solvents, it is possible that such treatments induce protein denaturation.

Hydroxyapatite (HA), Ca_10_(PO_4_)_6_(OH)_2_, is a major component of hard tissues such as bones and teeth and has been used as a biomaterial [5, 6]. Because it has been reported that some proteins, such as bovine serum albumin (BSA) and lysozyme, can bind to HA just by mixing, it is a good candidate for a protein carrier [7–9]. In this study, we investigated the association and dissociation behavior of two bioactive proteins, cytochrome c and insulin, to HA. It is known that the release of cytochrome c from mitochondria to the cytosol induces apoptosis. Therefore, the delivery of cytochrome c into the cytosol of cancer cells should induce apoptosis, which may be useful for cancer therapy [10]. Insulin, a key protein of diabetes, is commonly injected into diabetic patients to suppress blood sugar levels [11], and its controlled release can markedly improve their quality of life. Because the delivery of these two proteins is important, we attempted to use HA for a delivery system. The absorption and desorption on HA were affected by the surface conditions dependent on the preparation procedure of HA. Considering universal use of HA for protein delivery, commercially available HA was used as a carrier in this study.

## 2. Materials and Methods

HA nanoparticles were purchased from Sigma-Aldrich (MO, USA). According to the material data sheet (no. 677418), the size and surface area were smaller than 200 nm and larger than 9.4 m^2^/g, respectively. Cytochrome c and insulin were obtained from Nacalai Tesque Inc. (Kyoto, Japan). Physicochemical properties of proteins used in this study are listed in Table 1. Cytochrome c is cationic, and insulin is anionic at physiological pH, and the molecular weight of cytochrome c is larger than that of insulin.

Adsorption experiments with cytochrome c were performed by mixing HA (0, 10, 20, and 30 mg) with an aqueous solution (2.5 mg/mL, 200 *μ*l). After 4 h mixing with a rotator, centrifugation (15,000 g, 20°C, 60 min) was performed to collect the supernatants. These were analyzed by reversed phase high performance liquid chromatography (HPLC) to estimate the residual concentration of cytochrome c. The HPLC system was equipped with a cosmosil 5C18-MS-II column (Nacalai Tesque, Inc., Kyoto, Japan) and a UV detector (220 nm; UV-2075Plus, Jasco Inc., Tokyo, Japan). Samples (5 *μ*l) were injected with an autosampler (AS-2057Plus, Jasco Inc., Tokyo, Japan) and eluted with acetonitrile/0.05% trifluoroacetic acid = 20/80 (A) and acetonitrile/0.05% trifluoroacetic acid = 60/40 (B) at 1.0 mL*·*min^−1^ by PU-2089Plus (Jasco Inc., Tokyo, Japan). A linear gradient elution was performed over 20 min from an initial state (A) 100% to the final state (B) 100%. In the case of insulin adsorption, the same experimental procedures were performed except the insulin solution was prepared by dissolving it in 0.01 N HCl and adjusting to pH 3. Association ratio (%) was calculated as [(C_0_−C)/C_0_] ×100, at which C_0_ and C are the initial concentration and the supernatant concentration of proteins, respectively.****

During desorption experiments, HA (10 and 20 mg) absorbing cytochrome c and insulin was transferred into 400 *μ*l of phosphate buffer saline (PBS; 8 mM Na_2_HPO_4_, 2 mM KH_2_PO_4_, 137 mM NaCl, 3 mM KCl), and rotated. After predetermined incubation times, centrifugation (15,000 g, 20°C, 60 min) was performed to collect the supernatants. The residual concentrations of cytochrome c and insulin were estimated by HPLC. In the case of insulin, PBS adjusted to pH 3 was also used as the incubation buffer. Dissociation ratio (%) was calculated as [C/C_0_] ×100, at which C_0_ and C are the total concentration of the associated proteins and the supernatant concentration, respectively.

## 3. Results and Discussion

**Figure 1:**
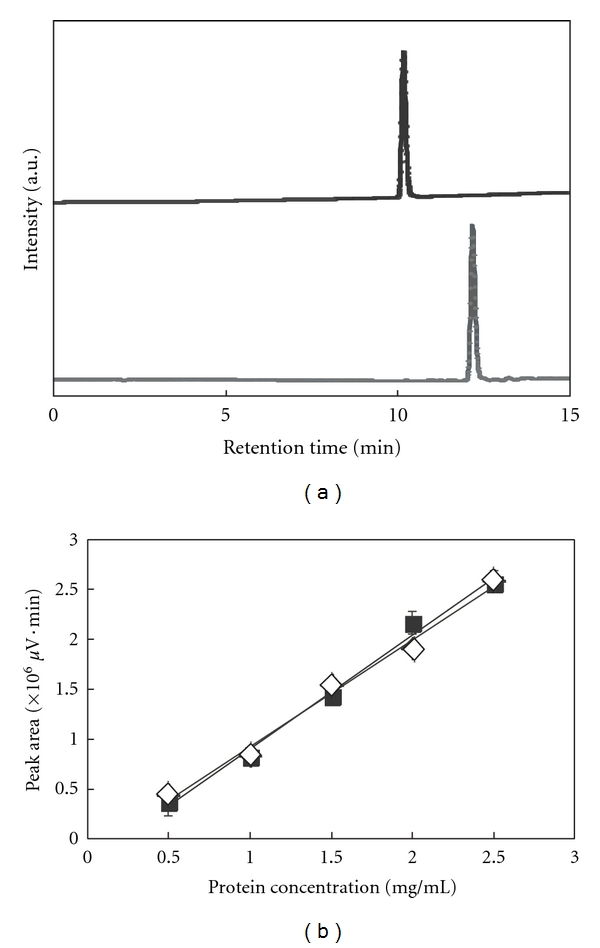
HPLC analysis. (a) Chromatograms of cytochrome c (top) and insulin (bottom). (b) Correlations between the peak area and the concentrations of cytochrome c (solid symbols) and insulin (open symbols).

**Figure 2:**
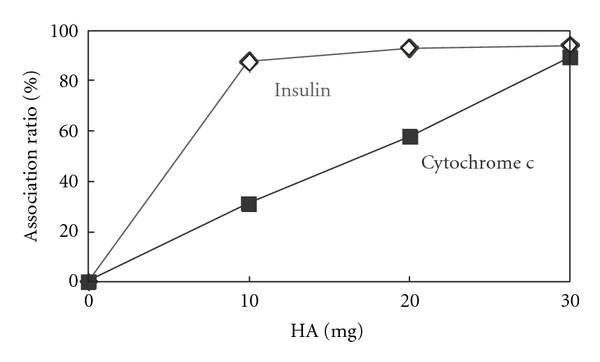
Association of cytochrome c (solid symbols) and insulin (open symbols) with various amounts of HA after 4 h.

The association experiments were performed by mixing HA and protein solutions. Cytochrome c was soluble in deionized water, but insulin was not. Therefore, insulin was dissolved in an acidic solution (pH 3). After the incubation and subsequent centrifugation, the residual cytochrome c and insulin in the solution were estimated from the HPLC analysis. Cytochrome c and insulin were eluted after 10 min and 13 min, respectively, under the running conditions (Figure 1(a)), and the peak areas were proportional to the protein concentrations (Figure 1(b)). Thus, the protein concentrations in the supernatants were evaluated by HPLC analysis and the adsorbed amounts were calculated by subtracting the concentrations in the supernatant from the initial ones.  Figure 2 shows the association ratio of these proteins on HA. Both proteins were associated with HA after the 4 h incubation. The adsorption efficiency of insulin was higher than that of cytochrome c. As less as 10 mg HA was sufficient to load almost 0.5 mg insulin, but 30 mg HA is necessary to load the same amount of cytochrome c under this condition. This may be because cytochrome c is larger than insulin. From the surface area of the HA (>9.4 m^2^/g) and the protein diameters (*d*) of insulin (3 nm) and cytochrome c (4 nm) [12], the percent of occupied area can be estimated. In the experiment, 0.5 mg of proteins, that is 9 × 10^-8 ^mol (5 × 10^16^ molecules) of insulin and 4 × 10^-8 ^mol (2 × 10^16^ molecules) of cytochrome c, were used. The projected area of proteins may be approximated as *π*(*d*/2)^2^, assuming the protein to be spherical. Given that all molecules were absorbed, the occupied areas of insulin and cytochrome c were 0.4 m^2^ and 0.3 m^2^, respectively. The surface area of HA (10 mg, 20 mg, and 30 mg) can be calculated as >0.094 m^2^, >0.19 m^2^, and >0.28 m^2^, respectively. It is suggested that the surface area of HA was fully occupied by proteins. Even though the absorption amount of cytochrome c could be correlated to the total surface area of HA, that of insulin could not. It is possible that insulin was absorbed to form multilayered structures.

**Figure 3:**
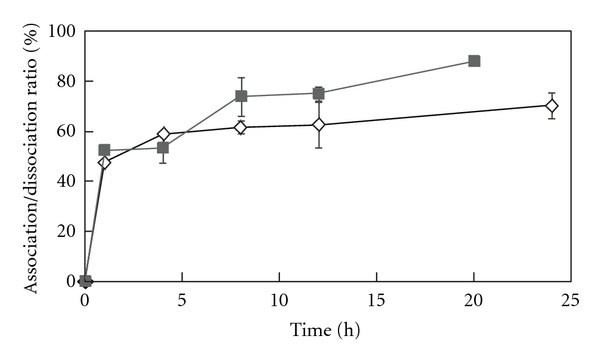
Time-dependent association (solid symbols) and dissociation (open symbols) of cytochrome c with HA (20 mg).

Figure 3 indicates time-dependent association of cytochrome c to HA. Long-term incubation contributed to the efficient loading of cytochrome c even with a small amount of HA. Figure 3 also indicates that cytochrome c bound to HA in two phases. The initial absorption occurred in less than 1 h, and the subsequent absorption occurred more slowly, in the range of an hour. The first absorption phase might be attributed to surface absorption and the latter by penetration into the pores. The release of cytochrome c was also examined at different incubation times. The release profiles also occurred over two phases, in less than 1 h and over the hour range (Figure 3), similar to the adsorption profile. This result suggests that absorbed proteins at the surface were released very fast and those within the pores were released more slowly. Thus, regulation of release can be achieved by the control of protein size and the pore size of HA. It is possible that step-by-step protein release can be performed in an HA-based delivery system.

**Figure 4:**
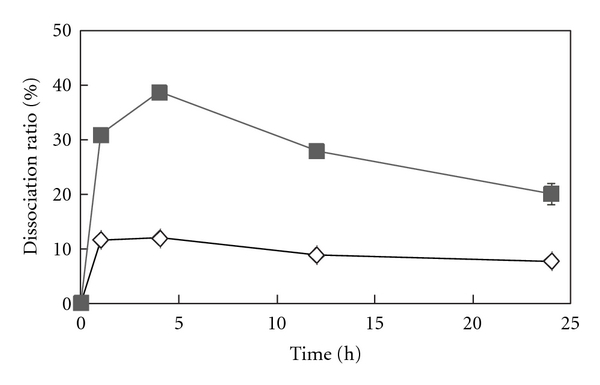
Time-dependent dissociation of insulin from HA (10 mg) at pH 7.4 (solid symbols) and pH 3.0 (open symbols).

Figure 2 shows that insulin was readily bound to HA. We also investigated the release profiles of insulin (Figure 4). Less than 40% of the bound insulin was released from HA, even though 70% of cytochrome c was released after 24 h. Insulin is smaller than cytochrome c and readily bound to HA (Table 1 and Figure 2), but the release of insulin is slower than that of cytochrome c (Figure 4). Therefore, this phenomenon was not related to protein size or absorption mechanism. It is likely that the slow release may be caused by its poor solubility in PBS, compared to cytochrome c. The release of insulin was next examined at pH 3, because insulin is easily soluble in acidic solution, which is a condition of the association. However, the release at pH 3 was slower than that at pH 7.4 and was perhaps affected by the charge of insulin. Insulin has an isoelectric point (pI) of 5.3 so is positively charged at pH 3 and negatively at pH 7.4. Hydroxyapatite is mostly negative, so cationic insulin might be more interactive with HA. A decrease in insulin release was observed, especially at pH 7.4 after more than 5 h. The readsorption of the released insulin to HA might have occurred, because the desorption conditions differed from the absorption condition. 

Our results suggest that the association and dissociation properties to HA were affected by both the charge and size of proteins. HA has a hexagonal structure, in which the C (Ca-rich) site is arranged in the a–c and b–c planes and the P (Ca-deficient) site is in the a–b plane. It was reported that anionic molecules bind to the C site and cationic ones to the P site [8]. Therefore, HA-based protein delivery is suitable for pH-dependent controlled release. Cationic cytochrome c and anionic insulin at the physiological pH were absorbed and desorbed in different manners. Because the charge of insulin was changed with decreasing pH, it markedly influenced the adsorption and desorption behaviors. The absorption behavior may be very complex, because large protein molecules bind to HA at multiple points. Therefore, the regulation of controlled release of protein is still to be investigated (For further information, see Supplementary Material available online at doi:10.1155/2012/932461.).

## 4. Conclusions

In conclusion, we prepared protein-associated HA and characterized its association and dissociation properties. Cytochrome c and insulin could bind to and release from HA. However, their association and dissociation behaviors differed, depending on the size and charge of the proteins. Therefore, HA is a potential carrier for protein delivery systems. 

## Supplementary Material

Hydroxyapatite (HA) has been studied as a biomaterial. We attempted HA to apply to delivery systems of bioactive proteins, such as cytochrome c and insulin. The association and dissociation properties of these proteins to HA were influenced by the size, solubility and net charge of protein. HA is a potential protein carrier with controlled release.

## Figures and Tables

**Table 1:** Proteins used in this study.

Protein	Mw (Da)	pI
Cytochrome c	12384	10.7
Insulin	5734	5.3

Mw: molecular weight, pI: isoelectric point.
