# Supplementary figures and images for: Adsorption and Desorption of Bioactive Proteins on Hydroxyapatite for Protein Delivery Systems
Source: J Drug Deliv. 2012 Mar 5;2012:932461. doi: 10.1155/2012/932461 (PMC3312276; doi:10.1155/2012/932461)

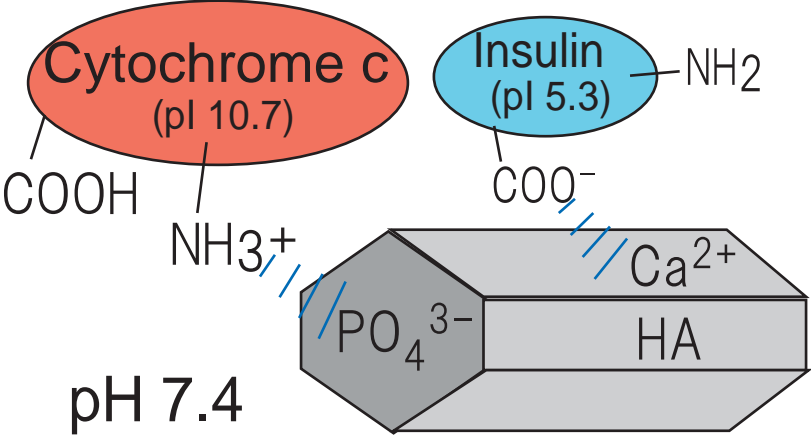

Supplement: Supplementary file 1 — Hydroxyapatite (HA) has been studied as a biomaterial. We attempted HA to apply to delivery systems of bioactive proteins, such as cytochrome c and insulin. The association and dissociation properties of these proteins to HA were influenced by the size, solubility and net charge of protein. HA is a potential protein carrier with controlled release. [file 932461.f1.pdf]
